# Comparison of chrysanthemum flowers grown under hydroponic and soil-based systems: yield and transcriptome analysis

**DOI:** 10.1186/s12870-021-03255-4

**Published:** 2021-11-08

**Authors:** Penghui Ai, Xiaoqi Liu, Zhongai Li, Dongru Kang, Muhammad Ayoub Khan, Han Li, Mengkang Shi, Zicheng Wang

**Affiliations:** 1 State Key Laboratory of Crop Stress Adaptation and Improvement, Plant Germplasm Resources and Genetic Laboratory, Kaifeng Key Laboratory of Chrysanthemum Biology, School of Life Sciences, Henan University, Jinming Road, Kaifeng, 475004 Henan China; 2 Zhengzhou A Boluo Fertilizer Company, Zhiji Road, Zhengzhou, 450121 Henan China

**Keywords:** Chrysanthemum tea, Hydroponic and soil system, Quality, RNA-seq analysis

## Abstract

**Background:**

Flowers of *Chrysanthemum* × *morifolium* Ramat. are used as tea in traditional Chinese cuisine. However, with increasing population and urbanization, water and land availability have become limiting for chrysanthemum tea production. Hydroponic culture enables effective, rapid nutrient exchange, while requiring no soil and less water than soil cultivation. Hydroponic culture can reduce pesticide residues in food and improve the quantity or size of fruits, flowers, and leaves, and the levels of active compounds important for nutrition and health. To date, studies to improve the yield and active compounds of chrysanthemum have focused on soil culture. Moreover, the molecular effects of hydroponic and soil culture on chrysanthemum tea development remain understudied.

**Results:**

Here, we studied the effects of soil and hydroponic culture on yield and total flavonoid and chlorogenic acid contents in chrysanthemum flowers (*C. morifolium* ‘wuyuanhuang’). Yield and the total flavonoids and chlorogenic acid contents of chrysanthemum flowers were higher in the hydroponic culture system than in the soil system. Transcriptome profiling using RNA-seq revealed 3858 differentially expressed genes (DEGs) between chrysanthemum flowers grown in soil and hydroponic conditions. Gene Ontology (GO) enrichment annotation revealed that these differentially transcribed genes are mainly involved in “cytoplasmic part”, “biosynthetic process”, “organic substance biosynthetic process”, “cell wall organization or biogenesis” and other processes. Kyoto Encyclopedia of Genes and Genomes (KEGG) analysis revealed enrichment in “metabolic pathways”, “biosynthesis of secondary metabolites”, “ribosome”, “carbon metabolism”, “plant hormone signal transduction” and other metabolic processes. In functional annotations, pathways related to yield and formation of the main active compounds included phytohormone signaling, secondary metabolism, and cell wall metabolism. Enrichment analysis of transcription factors also showed that under the hydroponic system, bHLH, MYB, NAC, and ERF protein families were involved in metabolic pathways, biosynthesis of secondary metabolites, and plant hormone signal transduction.

**Conclusions:**

Hydroponic culture is a simple and effective way to cultivate chrysanthemum for tea production. A transcriptome analysis of chrysanthemum flowers grown in soil and hydroponic conditions. The large number of DEGs identified confirmed the difference of the regulatory machinery under two culture system.

**Supplementary Information:**

The online version contains supplementary material available at 10.1186/s12870-021-03255-4.

## Background

Chrysanthemum (*Chrysanthemum* × *morifolium* Ramat.) originated in China and is a popular ornamental plant with a long history of cultivation [1]. It has been used for centuries as an important traditional Chinese tea, with reported anti-inflammation, antioxidant, and antiallergic properties [2, 3]. Traditional Chinese chrysanthemum tea is prepared by boiling chrysanthemum flowers in water to extract the beneficial compounds. Several studies have demonstrated that this chrysanthemum extract has strong antioxidant properties, and inhibitory effects against bacteria and viruses [3–7]. Flavonoids and chlorogenic acids have potent antioxidant activity and are among the biologically active components of chrysanthemum tea [2–8].

With the progress of industrialization and urbanization, land and water resources are increasingly scarce. Favorable environmental condition with quality sources such as soil and water are mainly supply the production of food crops. So being important economic crops, tea and medicinal chrysanthemum are in a disadvantageous state in terms of planting area and soil environment. Moreover, continuous cultivation of crops such as chrysanthemum in large areas can lead to soil-borne diseases, nutrient deficiency, and eventually declining yield and quality [9, 10]. Many studies have focused on different approaches to improve chrysanthemum yield and quality, including screening different varieties and developing new germplasm [2, 5, 11, 12], adjusting fertilizer ratios [13–15], testing the effect of abiotic stress [16], and examining the effect of different extraction methods on the activity of active compounds [3, 6]. However, these studies did not address issues associated with pressures on land use, or solve fundamental problems of soil and other aspects of the growth environment.

Hydroponic culture does not use soil, and so is not affected by soil-borne diseases or soil nutrient deficiencies. It also requires less water, fertilizer, and pesticides compared with cultivation in soil [17]. Changing the hydroponic medium enables adjustment of plant nutrients at any time. Hydroponic culture has been used for many cereal crops [18–22], fruits [22], vegetables [23–29], and flowers [30]. In some cases, hydroponic cultivation improves yield and quality [22–30]. Studies have explored the application of hydroponic technology to chrysanthemum, mainly to determine a suitable growth medium [30]. As a health food and tea, chrysanthemum is important for human disease resistance [3, 7, 31, 32]; therefore, the production of more chrysanthemum flowers with higher quality and less pesticide residues under controlled environmental conditions has become a top priority for chrysanthemum research. However, few studies have sought to understand the physiological and gene regulatory network differences of chrysanthemum grown in two culture systems.

Flower bud differentiation or development are important tissue to chrysanthemum for tea. Flowering is a complex process controlled by gene expression and phytohormones [33–40]. In chrysanthemum, important transcription factors (TFs) involved in flower development have been isolated and analyzed, including those encoded by the homologs of the Arabidopsis genes *APETALA1*, *SEPALLATA3*, *FRUITFULL*, *LEAFY*, *APETALA2*,*TEOSINTE BRANCHED1/CYCLOIDEA/PROLIFERATING CELL FACTOR20*, and *CYCLOIDEA2c*, as well as many genes encoding MADS-box and WUSCHEL (WUS)-like proteins [33–40]. In the current study, many members of different TF families, such as WRKY, MYB, MYC, NAC, C3H, Trihelix, C2H2, ERF and CRT TFs were upregulated during flower bud differentiation or development of chrysanthemum flower [35–40]. In sweet osmanthus (*Osmanthus fragras* Lour), low temperature increased the expression of genes related to cell expansion (encoding Expansin,

Xyloglucan endotransglycosylase/hydrolase, Xylosidase, Polygalacturonase) and phytohormones (Auxin, Gibberellic acid, Ethylene, and Brassinosteroids) to affect flowering time [41]. Transcription of expansin genes increased during flower opening of wintersweet (*Chimonanthus paraecox*) [42].

For foods with health-promoting properties, the level of bioactive compounds is an important component of quality. The flavonoid and chlorogenic acid biosynthetic pathways have largely been characterized [43]. Functional structural genes and regulatory gene pathways are conserved in plants [44]. Many functional genes (encoding chalcone synthase, chalcone isomerase, favone synthase, favanone 3-hydroxylase, favonoid 3′-hydroxylase, favonoid 3′,5′-hydroxylase, favonol synthase, dihydrofavonol 4-reductase, anthocyanidin synthase and UDP-glucose-favonoid 3-O-glucosyltransferase) in the flavonoid biosynthetic pathway have been identified in chrysanthemum. Additional, in *C. morifolium* ‘Chuju’, MYB–bHLH–WD40 TF complexes regulate flavonoid biosynthesis during the beginning of flower bud formation [44].

In recent years, RNA-seq has been used for transcriptomic analysis of flower development [35–39, 43], secondary metabolite synthesis [44–46], and stress tolerance [47–49] in chrysanthemum and its wild relatives [50, 51]. Such analysis has helped to reveal the molecular mechanisms of various processes and discover many candidate genes (homologs of *Early Flowering, Late Elongated Hypocotyl, Pseudo-Tesponse Regulators, Circadian Clock Associated, Chalcone Synthase, Chalcone Isomerase, Favone Synthase, Favanone 3-hydroxylase, Favonoid 3′-hydroxylase, Favonoid 3′,5′-hydroxylase, Favonol Synthase, Dihydrofavonol 4-reductase, Anthocyanidin Synthase and UDP-glucose-favonoid 3-O-glucosyltransferase,*et al.*,)* for various type of conditions. However, chrysanthemum has many hybrid polyploid varieties that lack high-quality reference genome information. Fortunately, whole-genome fine mapping of *C. indicum* ‘Nankingense’ has been established using next-generation sequencing [52]. *C. indicum* is a diploid plant and a wild ancestor of *Chrysanthemum* × *morifolium* (Ramat.) [1]. Whole-genome fine mapping of this variety provides a reliable reference genome for transcriptome analysis of chrysanthemum.

Hydroponic culture can improve the quantity or size of fruits, flowers and leaves, as well as increase production of the nutritionally and medicinally important active compounds in many crops, fruits, vegetables, and flowers [22–31]. Therefore, the transcription of genes related to flower development and active compound biosynthesis might be upregulated in response to hydroponic culture. To test this, in the current study, we used RNA-seq to identify differentially expressed genes (DEGs) involved in metabolic pathways in two culture systems. Identification of DEGs in chrysanthemum may provide new genetic resources for this species. This study will help reveal the molecular mechanisms underlying chrysanthemum yield and qualities associated with hydroponic culture.

## Results

### Comparison of hydroponic and soil-grown chrysanthemum

**Fig. 1:**
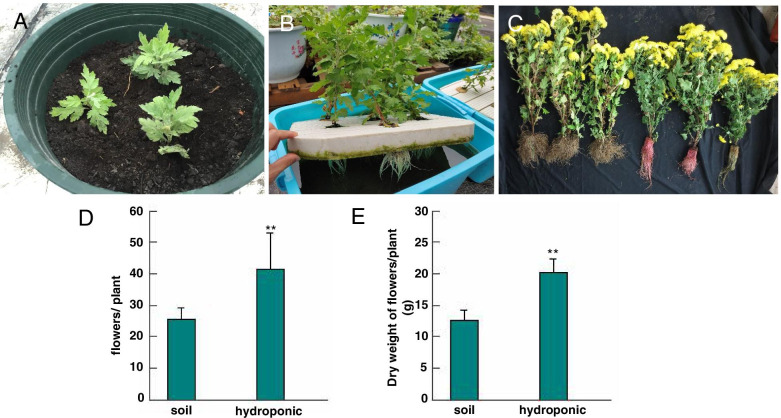
Phenotype, number and dry weight of chrysanthemum under soil system and hydroponic system. **A** Phenotype of chrysanthemum at seeding stage under Soil cultivation; **B** Phenotype of chrysanthemum at seeding stage under Hydroponic cultivation; **C **Phenotype of chrysanthemum at blooming stage under Soil system(left) and hydroponic system(right); **D** Comparative number of flowers from one plant of chrysanthemum grown under soil system and hydroponic system.*P < 0.05, **P < 0.01; **E** Comparative dry weight of flowers from one plant of chrysanthemum grown under soil system and hydroponic system. *P < 0.05, **P < 0.01

Hydroponically cultivated chrysanthemum plants grew normally (Fig. 1A, B, C). When harvested for comparison (Fig. 1D, E), the average number and dry weight of hydroponically cultivated chrysanthemum flowers were greater than field-cultivated ones (41.33 ± 11.64 flowers per plant, and 20.25 ± 5.70 g dry weight per flower for hydroponic cultivation versus 25.67 ± 3.43 flowers per plant, and 12.58 ± 1.68 g dry weight for soil cultivated flowers, meaning increased production by up to 37.89% with hydroponic cultivation).

**Fig. 2:**
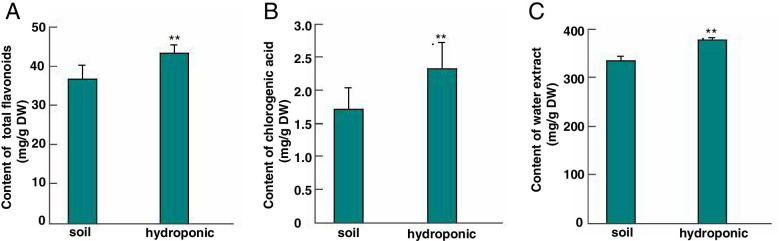
Comparative characterization of chrysanthemum grown by hydroponic system and soil system for flower quality. **A** Difference of total flavonoids content in chrysanthemum flower under soil and hydroponic system. *P < 0.05, **P < 0.01; **B** Difference of total chlorogenic acid content in chrysanthemum flower under soil and hydroponic system. *P < 0.05, **P < 0.01; **C** Difference of total water extract content in chrysanthemum flower under soil and hydroponic system. *P < 0.05, **P < 0.01

Chrysanthemum tea extracts from flowers grown under soil or hydroponic systems showed differences in their biologically active components. Hydroponically cultivated chrysanthemum flowers water extracts achieved 37.8% ± 4.47, which was higher than soil-cultivated chrysanthemum flowers (33.4% ± 10.45) (Fig. 2C). Total flavonoids and chlorogenic acid content of chrysanthemum flowers grown in soil and hydroponic systems are shown in Fig. 2A, B. Flowers grown hydroponically had higher flavonoid and chlorogenic acid contents (43.22 ± 2.05 mg/g and 2.330 ± 0.40 mg/g dry weight [DW], respectively) than flowers grown in soil (36.75 ± 3.50 mg/g and 1.715 ± 0.32 mg/g DW, respectively).

### RNA-seq analysis of chrysanthemum flowers grown in hydroponic and soil systems

To better understand how different culture systems affect molecular mechanisms in chrysanthemum flower, total RNA from chrysanthemum flowers grown in soil and hydroponic systems was sequenced using an Illumina system. Transcriptomic data of six samples were obtained, with three biological replications for each condition. RNA-seq analysis provided 41.9–47.5 million raw reads per biological replicate, with an average read length of 100 bp (Table 1). After filtering out adapters, low-quality reads, virus reads and unblasted reads, approximately 133.1 million clean reads were obtained across the six transcriptome libraries. Q20 percentages of each sample (sequencing error rates lower than 1%) were higher than 98.17%. Meanwhile, 77.69–87.09% of clean reads could be aligned with the *C. nankingense* reference genome [53], 71.89–80.52% could be accurately mapped to a specific location within the reference genome sequence, and 5.78–6.57% mapped to multiple locations. The gene expression level could be quantified by normalized fragments per kilobase of transcript per million mapped reads (FPKM) [53]. FPKM data were tested to evaluate correlations among biological replicates; all the Pearson correlation coefficients among biological replicates were higher than 0.95 (Figure S1). In addition, principal component analysis (PCA) was performed to visualize the variation among samples utilizing DESeq2 [54], and the biological replications for each sample were clustered together (Figure S2). Overall, the transcriptomic data in this work were viable on the basis of statistical analysis from chrysanthemum flower RNA sequencing.

**Table 1:** Summary of the sequence data from RNA sequencing

Sample	Raw Reads	Clean Reads	Virus Reads and unblasted Reads	Effective Reads	Q20(%)	Mapping Ratio(%)	Multiple Mapping Ratio(%)	Mapped Reads
Flower-CK-1	43556646	43470330	19475428	23994902	98.24	77.33	6.12	20020426
Flower-CK-2	45390350	45340972	21579690	23761282	98.25	77.18	6.08	19785119
Flower-CK-3	47512154	47472814	28923258	18549556	98.24	72.73	5.78	14561962
Flower-SP-1	44441130	44392748	26520674	17872074	98.18	71.86	5.83	13884621
Flower-SP-2	41878816	41826132	23494042	18332090	98.17	72.43	5.84	14347441
Flower-SP-3	45985172	45045678	15383196	30562482	98.24	80.52	6.57	26614315

### Identification and analysis of DEGs

**Fig. 3:**
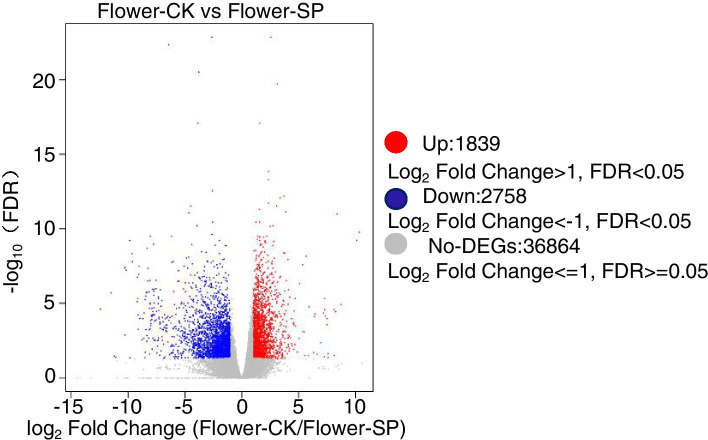
The expression profile of DEGs in flowers of chrysanthemum under two culture systems

Chrysanthemum flowers grown under soil and hydroponic systems were compared to identify DEGs (*q*-value≤0.05 and |log_2_FC| ≥ 1) by DEGseq2 software. A total of 3858 genes were differentially expressed in chrysanthemum flowers grown under soil and hydroponic systems, with 1556 upregulated (Table S5) and 2306 downregulated in plants grown soil compared with hydroponically-grown plants (Fig. 3A, Table S6).

**Fig. 4:**
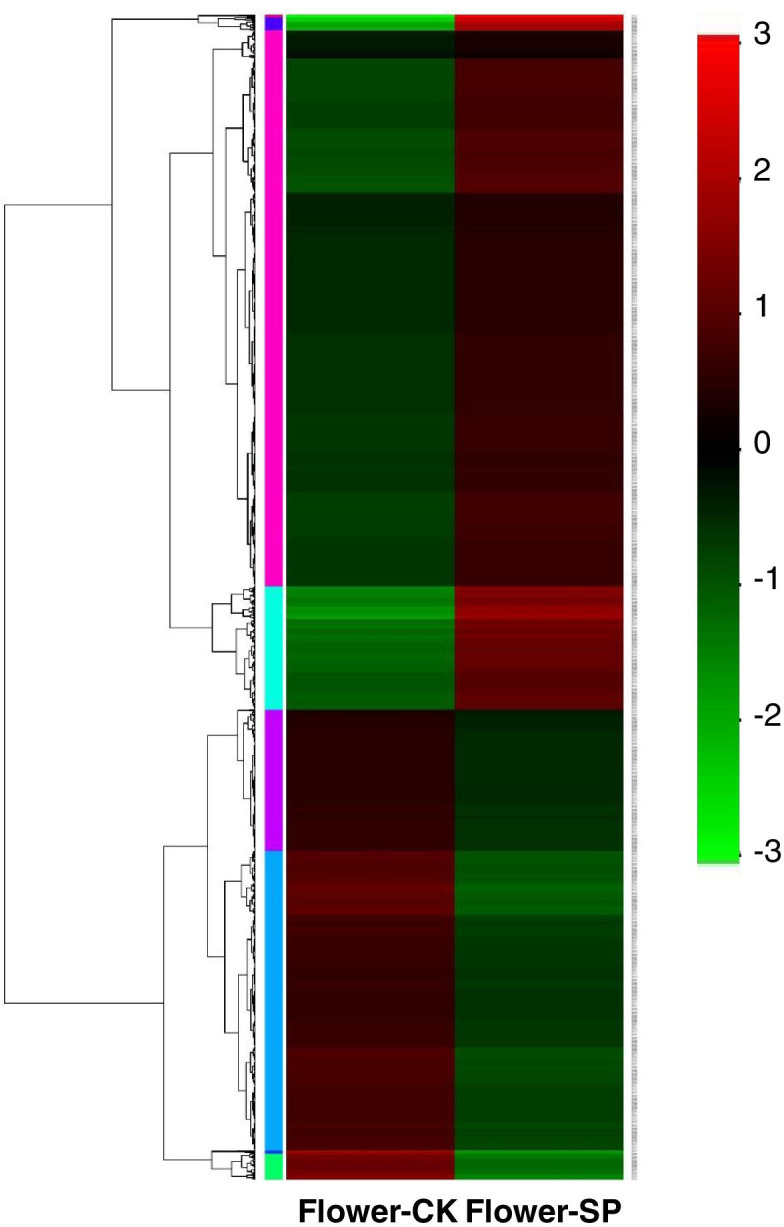
Hierarchical cluster analysis of 3858 DEGs based on the log (FC) of gene expression. The color gradient from low (blue) to high (red) represents relative levels of gene expression. The numbers in the scale bar stand for the score of gene expression

Based on hierarchical clustering analysis of gene expression, 3858 DEGs could be roughly grouped into four classes (Fig. 4). A total 104 DEGs were specifically expressed in chrysanthemum flowers grown in the hydroponic system, including many genes involved in “carbohydrate metabolic” (GO:0005975), “transcription” (GO:0006351), “fatty acid and unsaturated fatty acid biosynthetic process” (GO:0006633, GO:0006636), “auxin-activated signaling pathway” (GO:0009734), and “phenylpropanoid metabolic process” (GO:0009698). These genes may have important functions in chrysanthemum flower development. Conversely, ten DEGs were specifically expressed in chrysanthemum flowers grown in the soil system, including genes involved in “transcription” (GO:0006351), and “protein phosphorylation” (GO:0006468). More unigenes were downregulated than upregulated in plants grown soil compared with hydroponically-grown plants.

### GO and KEGG enrichment analysis of DEGs

**Fig. 5:**
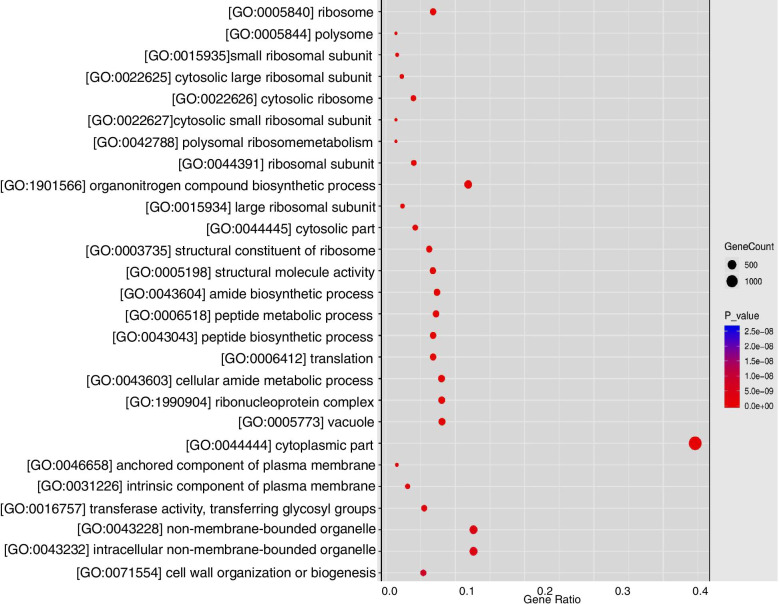
Categories and distribution of GO terms in the flowers under soil and hydroponic system

To determine the fundamental functions of the obtained DEGs, GO analysis was performed using Cluster Profile R packages [55]. A total of 3858 DEGs were enriched in 266 GO terms, and could be classified into three categories: biological process, molecular function, and cellular component. In particular, there were 62 GO terms in which DEGs were enriched by a factor of more than 100 (Table S1). GO annotation revealed 62 GO terms for 29 annotations in cellular components, 27 annotations in biological processes, and 6 annotations in molecular functions. “Cytoplasm” (GO:0005737), “cytoplasmic part” (GO:0044444), “membrane” (GO:0016020), “cell periphery” (GO:0071944), and “cell wall” (GO:0005618) were significantly enriched in the cellular components category. In the biological processes category, “biosynthetic process” (GO:0009058), “organic substance biosynthetic process” (GO:1901576), “cellular biosynthetic process” (GO:0044249), and “cell wall organization or biogenesis” (GO:0071554) were enriched. In the molecular functions category, major enriched functions were “transmembrane transporter activity” (GO:0022857), “structural molecule activity” (GO:0005198), “transferase activity, transferring glycosyl groups” (GO:0016757), and “structural constituent of ribosome” (GO:0003735). Furthermore, the enriched GO terms from flowers grown under two culture systems were compared on the basis of their biological processes (Fig. 5).

**Fig. 6:**
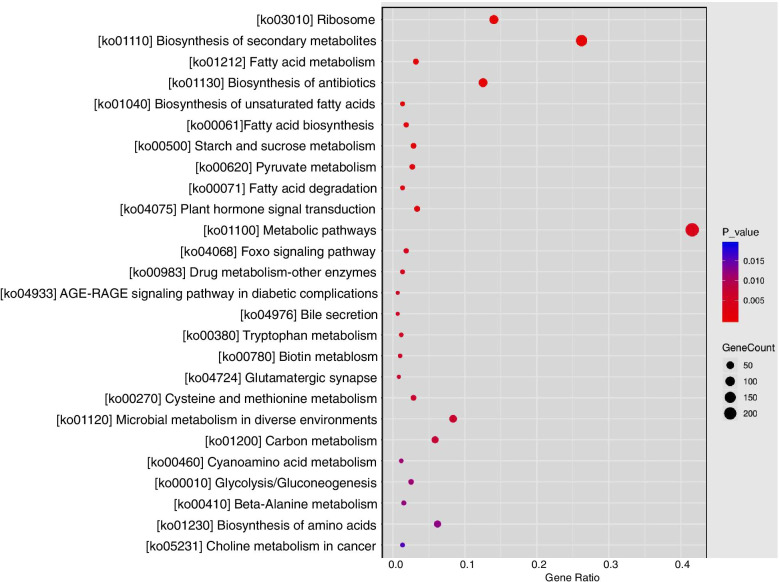
KEGG pathways prominently enriched DEGs in the flowers under soil and hydroponic system

To identify the metabolic pathways of genes differentially expressed in the hydroponic system, we first searched the DEGs against KEGG using KofamKOALA. A total of 3858 DEGs were anchored in KEGG Orthology (KO) terms, wherein 873 DEGs were prominently enriched in 25 pathways (Fig. 6, Table S2). It is noteworthy that 246 and 160 DEGs were found enriched in two pathways, “metabolic pathways” (ko01100) and “biosynthesis of secondary metabolites” (ko02220). Furthermore, four pathways, “biosynthesis of secondary metabolites” (ko02220), “starch and sucrose metabolism” (ko00500), “plant hormone signal transduction” (ko04075) and “fatty acid degradation” (ko00071) include up- and downregulated DEGs.

### Identification of DEGs involved in secondary metabolism

**Fig. 7:**
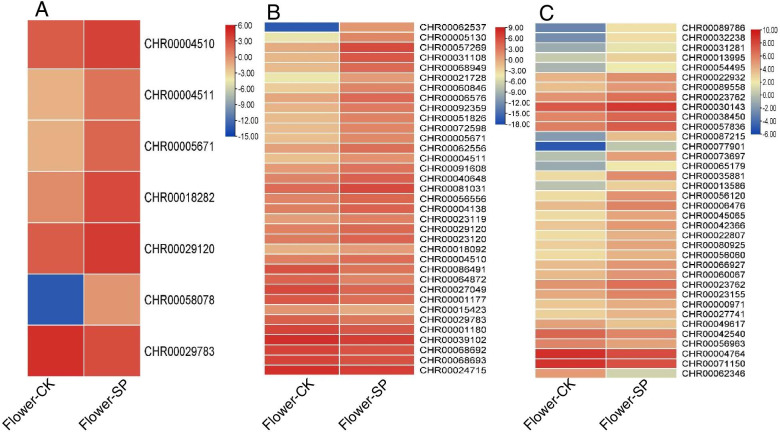
Heat maps of gene expression patterns involved in biological pathways obtained from KEGG. Heat maps were drawn according to log2FPKM in flowers under soil and hydroponic system. The rows and columns in the heat maps represent samples and genes id, respectively. Red and blue represent the highest and lowest level of expression. **A** Flavonoid biosynthesis; **B** phenylpropanoid biosynthesis; **C** fatty acid biosynthesis

Chrysanthemum contains many bioactive ingredients: flavonoids, triterpenes, sterols, volatile oils and fatty acids, which are beneficial to humans. We focused on genes involved in “flavonoid biosynthesis” and “fatty acid and unsaturated fatty acid biosynthesis”. KEGG annotation results revealed many biosynthesis processes including “flavonoid biosynthesis”, “phenylpropanoid biosynthesis” and “fatty acid and unsaturated fatty acid biosynthesis” (Table S2), which were significantly enriched in flowers grown under the hydroponic system. Among key genes in the flavonoid pathway, shikimate O-hydroxycinnamoyltransferase, caffeoyl-CoA O-methyltransferase, chalcone-flavonone isomerase, and dihydroflavonol 4-reductase were the most upregulated, while caffeoyl-CoA O-methyltransferase was downregulated in flowers grown under hydroponic system conditions (Table S3, Fig. 7A). Phenylpropanoid synthesis is important since it is the initial substrate of many secondary metabolites. Phenylpropanoid biosynthesis was also enriched with many DEGs, including 24 up- and 11 downregulated (Table S4 Fig. 7B). Thirty-seven genes were identified in the fatty acid and unsaturated fatty acid biosynthesis pathway, of which 30 were significantly upregulated and 7 were significantly downregulated (Table S5, Fig. 7C).

### Identification of DEGs involved in cell wall metabolism

**Fig. 8:**
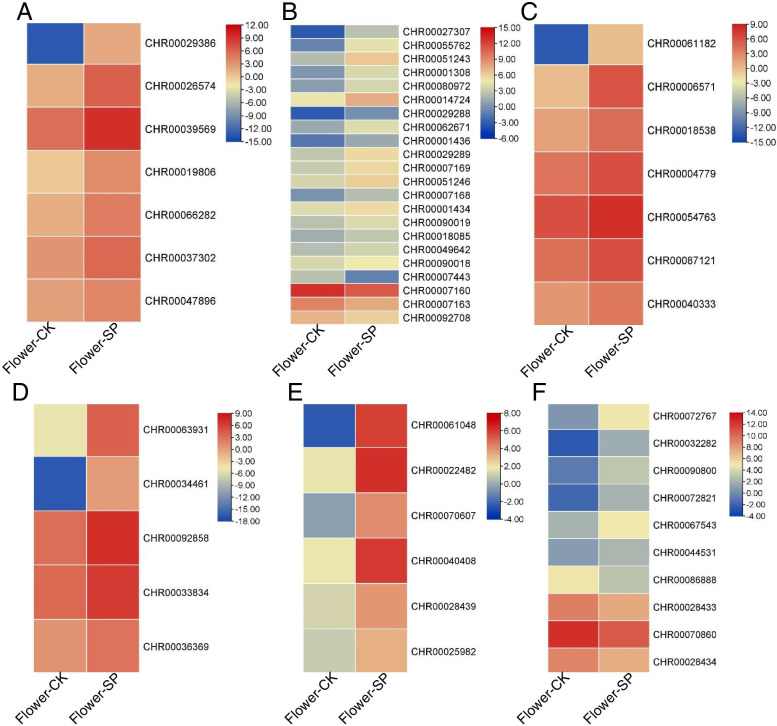
Heat maps of cell wall metabolism related genes in flowers. Heat maps were drawn according to log_2_FPKM in flowers under soil and hydroponic system. The rows and columns in the heat maps represent samples and genes id, respectively. Red and blue represent the highest and lowest level of expression. **A** EXP: expansin gene, **B** PE: pectinesteraseg gene; **C** PL: pectate lyase gene; **D** PG: polygalacturonase gene; **E** XTH: xyloglucan endotransglucosylase/hydrolase gene; **F** XYL: xylosidase gene

Flower growth depends on cell wall loosening and cellulose biosynthesis, soluble carbohydrate allocation and cytoskeleton rearrangement [56, 57]. Therefore, we screened for DEGs that might be involved in cell wall metabolism under different culture systems. In our study, seven expansin genes, six xyloglucan endotransglucosylase/hydrolase genes, five polygalacturonase genes and seven pectate lyase genes were upregulated under hydroponic conditions, suggesting that these genes are important for regulating chrysanthemum flower growth. Four xylosidases were down-regulated undrer soil conditions and 6 of them up-regulated. Eighteen pectinesterase were up-regulated under hydroponic conditions except CHR00007443, CHR00007160, CHR00007163 and CHR00092708 (Table S6, Fig. 8).

### Transcription factor upregulation occurred more in hydroponically grown flowers

**Fig. 9:**
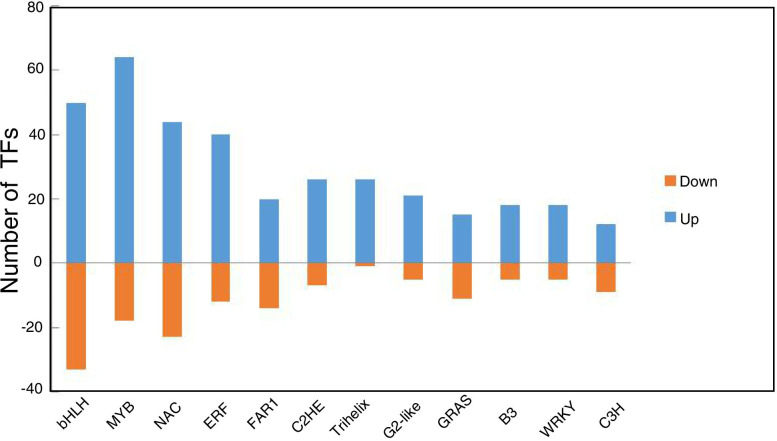
Determination of the top 12 transcription factor families in flowers under soil and hydroponic system. Blue represents the up-regulated TFs, yellow represents the down-regulated TFs, and the vertical axis shows the number of TFs

We further analyzed TFs to better understand the regulatory network of chrysanthemum flowers grown under both culture systems. Transcriptome sequencing results showed that 2298 and 1580 TFs were up- and downregulated, respectively, in flowers under hydroponic conditions, and these were classified into 52 transcription factor families. Expression of many TFs changed dramatically under different growth systems, so we improved the screening criteria. TFs with a |log_2_Ratio| ≥ 2 in any comparison were analyzed further (Table S7). The highest number of TFs belonged to the bHLH (83, 10.64%), MYB (82, 10.51%), NAC (67, 8.59%), and ERF (52, 6.67%) families. Figure 9 shows bar graphs of the top 12 transcription factor families and a comparison of up- and downregulated TFs in flowers grown under soil and hydroponic conditions. A small proportion of TF genes were downregulated under hydroponic conditions. These important TF genes were substantially differentially expressed between the two growth conditions, and may be crucial for the differences in flower development.

### Validation of RNA-seq analysis by qRT-PCR

**Fig. 10:**
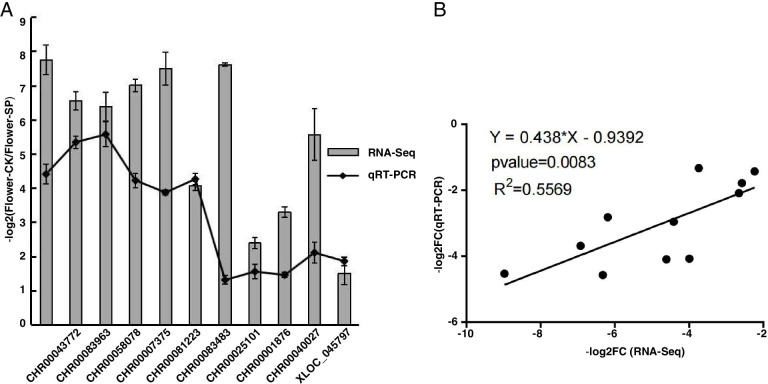
Verification of RNA-Seq results by qRT-PCR. **A** Relative expression ratio of each DEG is presented in -log2(Fold Change). **B** Correlation between RNA-Seq and qRT-PCR

To determine the reliability of DEGs obtained from RNA-seq analysis of chrysanthemum flowers, 11 DEGs were selected for qRT-PCR analysis that participated in different biological pathways including “flavonoid biosynthesis”, “plant hormone signal transduction”, “photosynthesis” (Table S8), wherein *GAPDH* was used as the reference gene for normalization. As a result (Fig. 10), high correlation (R^2^ = 0.557) between qRT-PCR and RNA-seq was observed. This result unambiguously confirmed the reliability of the DEGs obtained from RNA-seq analysis in this study.

## Discussion

Many plants can grow in hydroponic systems [17–30]. For crops, the quality of produce, taste and the nutritive value of the end products is generally higher in hydroponic systems than in natural soil cultivation. Hydroponic culture can avoid problems such as the reduction of high-quality land area, shortage of fresh water resources, and reduction of yield caused by soil-borne diseases. There has been much research into how hydroponic culture systems affect plant growth and development, but their effect on growth, development, and regulation of molecular network mechanisms remains unclear for chrysanthemum flower.

In the present study, yield and quality of chrysanthemum flowers grown under a hydroponic culture system were higher than those grown in a soil system. RNA-seq technology was used to explore the effects of hydroponic culture on chrysanthemum flower. Transcriptome sequencing was used to analyze the flower, leaf and root under light, salinity and other conditions of chrysanthemum, and many key genes were also found [35–51]. Through KEGG and GO enrichment analysis, key genes and biological pathways were screened to comprehensively analyze the effects of hydroponic culture on gene expression and regulatory networks. The identification of DEGs in this species may provide new genetic resources for improving yield and quality of chrysanthemum.

### Growth and development regulation of flowers

In this study, we analyzed the number and dry weight of chrysanthemum flowers grown in different culture systems. Our results indicated that the hydroponic system was more efficient for growing chrysanthemums than soil culture. Flower growth is accompanied by cell division and cell expansion in petal cells [56, 57]. Cell wall synthesis, modification or hydrolysis; cell expansion and loosening; and rearrangement of cell wall fibers in growing tissues probably participate in the process of flower growth [58–60]. Similarly, during growth of chrysanthemum flowers in a hydroponic system, seven expansin genes, six xyloglucan endotransglucosylase/hydrolase genes, five polygalacturonase genes, and seven pectate lyase genes were upregulated, and partial xylosidase, cellulose synthase, and pectinesterase genes were downregulated (Table S6, Fig. 7). Upregulation of several genes suggests that these genes may be involved in petal development in hydroponic systems.

Floral organ morphogenesis and growth are also influenced by multiple phytohormones. ARF6 and ARF8 induce jasmonate production, which in turn triggers expression of MYB21 and MYB24, which promote petal and stamen growth in *Arabidopsis thaliana* [61]. Gibberellic acid and brassinosteroids promote petal development; however, abscisic acid has an antagonistic role in petal development [62, 63]. The expression patterns of phytohormone pathway genes are different under different culture systems. Most DEGs involved in the auxin, jasmonate and brassinosteroid signaling pathways were upregulated (Table S9); however, most DEGs involved in the abscisic acid pathway were downregulated in the hydroponic system (Table S9). This suggests that effects of the hydroponic system on chrysanthemum flower growth may be dependent on phytohormone signaling.

One beneficial effect of hydroponic systems is improved photosynthesis and reduction of the portion of fixed CO_2_ released into the rooting medium as non-respiratory carbon [64]. The conventional conception of most white or colored flower petals is that they are composed of non-photosynthetic tissues that lack chlorophyll. However, Lysenko and Varduny (2013) found that colored flower petals of *Petunia hybrida* that lack chlorophyll are capable of light energy storage, as well as usual chlorophyll photosynthesis accompanied by additional ATP synthesis [65]. In this experiment, DEGs involved in photosynthesis pathways exhibited more obvious differences between the two culture systems. DEGs encoding chlorophyll a-b binding protein, dihydrodipicolinate reductase-like protein CRR1, psbP-like protein 2, photosystem II PsbW, psbQ-like protein, PDZ domain, magnesium-chelatase subunit ChlI protein, and magnesium chelatase subunit H of important photosynthesis-related genes were upregulated in flowers grown in the hydroponic system (Table S10). Whether chrysanthemum flowers photosynthesize like leaves requires further research; however, these results suggest that hydroponic culture can reduce the expression of some important photosynthesis-related genes.

### Biosynthesis of flavonoids, chlorogenic acids, and other metabolites

Epidemiological studies indicate that chronic inflammation and oxidative stress are critical in neoplastic development [66]. Some currently used synthetic free radical scavengers have demonstrated various side effects [67, 68]. Therefore, functional foods and teas have become a promising source of natural antioxidants [66]. Chrysanthemum has a long history as an herbal tea or medicine for the treatment of various inflammatory diseases. Many studies have shown that chrysanthemum has multiple activities, with antioxidant activity being of particular interest [2–7]. Therefore, improving the accumulation and activity of secondary metabolites in chrysanthemum has become a hot topic of research.

Chrysanthemum secondary metabolites, such as flavonoids and chlorogenic acid, are non-enzymatic antioxidants. Flavonoid and chlorogenic acid biosynthetic pathways have largely been characterized [43], and have been shown to affect many conditions, such as high and low mineral nutrition [69–72], drought [16, 73–75], heat [75], cold [76], high salinity [77], and other environmental stresses [75, 78]. In the present study, total flavonoid and chlorogenic acid concentrations in flowers grown in a hydroponic system were higher than those grown in a soil system. Interestingly, transcript levels of the DEGs *CHR00004510*, *CHR00004511*, *CHR00005671*, *CHR00018282*, *CHR00029120*, *CHR00058078*, which encode key genes of the phenylpropanoid pathway, were also upregulated in flowers cultured under a hydroponic system (Fig. 7A).

In the current study, low nitrogen treatment given after the chrysanthemum bud differentiation stage increased total flavonoids, chlorogenic acid contents, and the content of other secondary metabolites without affecting yield. Nitrogen content was lower in the hydroponic system than in the soil system (Figure S4). Our findings are also consistent with previously reported studies [69–71]. Soil can absorb and fix nutrients that plants are unable to effectively absorb, or are unable to absorb at a given time. However, hydroponic systems allow plants to rapidly take up nutrients and the nutrient content of plants may be altered by changing the nutrient solutions. This simple and effective method provides us with a new way to cultivate chrysanthemums with high levels of calcium, iron and selenium, among others.

The total flavonoid content of extracts from the chrysanthemum variety “Wuyuanhuang” was 36.75–43.22 mg/g DW (Fig. 2). This was lower than extract of “Kunlun XueJu” (87.2 mg/g DW), which was higher than “Huang Ju”, “Hangbai Ju”, “Tai Ju”, “Gong Ju”, and “Ganye Ju”. Chlorogenic acid content of extracts from the chrysanthemum variety “Wuyuanhuang” was 1.71–2.33 mg/g DW, which was higher than “Kunlun XueJu” and “Ganye Ju” [2]. Thus, *C. morifolium* ‘Wuyuanhuang’ is a good chrysanthemum tea variety. Agreeing with previous studies, our results indicate that individual chrysanthemum samples and different extraction methods might significantly differ in their flavonoid and chlorogenic acid compositions [2, 4, 6, 7]. Although we did not investigate whether hydroponic systems can affect the antioxidant activity of the flowers, previous studies have demonstrated a significant, positive correlation between the antioxidant activity of *C. morifolium* flowers and the concentrations of total flavonoids and chlorogenic acid [79].

### Transcription factors

Comparative transcriptome analysis revealed a wealth of information. Many important TFs were significantly upregulated, and a few TFs were turned off in chrysanthemum flowers cultivated in a hydroponic system (Fig. 9). Transcription factors make important contributions to the regulation of plant growth and development, and can also influence plant secondary metabolites through orchestrating regulatory networks [33–44]. The C2H2 zinc finger transcription factors are reportedly involved in many biological processes related to plant or floral growth and development, hormone signaling, and stress responses in Arabidopsis and chrysanthemum [37, 38, 80]. In the present study, 26 and 7 C2H2 genes were up- and down-regulated, respectively, in flowers grown in a hydroponic system, implying that C2H2 TFs are important for chrysanthemum flower development or the biosynthesis of flavonoids and chlorogenic acid.

MYB and bHLH transcription factors are important regulators involved in controlling plant or flower growth and development, and are involved in secondary metabolites synthesis in plants [81–84]. Notably, MYB–bHLH–WD40 complexes modulate flavonoid biosynthesis in *C. morifolium* ‘Chuju’ [44]. In this work, many unigenes homologous to MYB and bHLH were identified. Sixty-three and 18 MYB genes, and 50 and 33 bHLH genes were up- and downregulated in flowers grown under a hydroponic system, respectively. One MYB and two bHLH genes were not expressed in flowers grown in soil. Accordingly, functional characterization of these genes is required under hydroponic conditions.

Comparative transcriptome analysis found one WD40 gene was upregulated in flowers grown in a hydroponic system (Table S7). The FACTOR (TCP20) transcription factor of chrysanthemum is reportedly involved in regulation of petal size by interacting with CmJAZ1-like and inducing downregulation of *CmBPE2* gene expression [34]. Transcriptome analysis found three TCP TFs (one *CYC4* gene) were upregulated in flowers under hydroponic conditions. In this work (Fig. 9), ERF, Trihelix, WRKY and C3H family members were identified in chrysanthemum, and were upregulated during chrysanthemum flower bud differentiation or development [35–41]. This implies that these genes may be involved in flower developmental processes, and these TFs should be further studied in chrysanthemum. Overall, TFs were upregulated more than downregulated in flowers grown under a hydroponic system, implying that flowers grown hydroponically have more active genes than those grown in soil. This may be why hydroponically grown chrysanthemum flowers have improved yield and active compounds compared with those grown in soil.

## Conclusions

In the present study, the yield of chrysanthemum flowers, and total flavonoids and chlorogenic acid of chrysanthemum tea made from flowers grown in a hydroponic culture system, were higher than those grown in a soil system. Transcriptome analysis indicated that DEGs between flowers grown under these two culture systems were enriched in GO and KEGG pathways relating to the regulation pf flower growth and development, and secondary metabolism. Our results will contribute to knowledge of effective and simple cultivation methods for chrysanthemum tea, and identify genes involved in mechanisms influenced by hydroponic systems for improving chrysanthemum yield and total flavonoids and chlorogenic acid.

## Methods

### Plant materials

All trials were carried out with chrysanthemum stock plants (‘Wuyuanhuang’) that is a Chinese traditional tea Chrysanthemum. All plant materials were owned from our laboratory, Plant Germplasm Resources and Genetic Engineering, Henan University, for 10 years. Chrysanthemum plating experiment was conducted at the Zhenghzhou A Boluo fertilizer company (113°43′N, 34°76′W) that was our unit from June 1 to November 92,020. The rooted young plants were fixed on the polyethylene lay and floated in nutrient solution for cultivation and growth. For comparison, the same Chrysanthemum plants cultivated in soil as control (Fig.1A, B). The initial physico-chemical properties of soil in this experiment, such as pH (8.1), soil nitrogen concentrations (0.97 g/kg), soil available phosphorus concentrations (7.6 mg/kg), soil rapidly available potassium concentration (106 mg/kg) came from the company farm. The experiments were carried out in outdoor. The nutrient solution composition was as: A Boluo chrysanthemum self-fertilizer (nitrogen, phosphorus, potassium and trace elements) 10 g / 20 L water, pH adjusted as 5.8–6.0, EC was 1.0–1.3 ms/ cm. During the seedling stage, water was added every day to maintain the normal supply (loss of water cause by evaporation due to high temperature) and determine EC weekly. Adding fertilizer adjusted the solution to 1.0–1.3 ms/cm. Nitrogen treatment was stopped when plants entered the full bloom stage. Harvesting took place after a week of nitrogen stoppage. The soil cultivated chrysanthemum plants were treated with the same amount of fertilizer as hydroponic system.

All flowers of each plant were collected and dried at 40–45 °C in a forced-air oven system according to statistic quantity and weight caliber (Fig. 1D, E). The flowers were pulverized and stored at − 25 °C for quality analysis.

### Measurement of total flavonoid, chlorogenic acid and water extracts concentration

Total flavonoid was measured as described by Dahui Liu (2010) [70]. Briefly, 1.0 g powered sample of flowers were extracted with 60 mL 70% ethanol in shaker at 60 °C for 30 min,160 r/min speed, then filtered and added 70% ethanol to 100 mL. One milliliter of the ethanol extract was added to the test tube, followed by the addition of 4 mL 60% ethanol and 0.5 mL 5 g/100 mL NaNO_2_ solution. After 5 min, 0.5 mL 10 g/100 mL Al (NO_3_)_3_ solution and 4 mL 10 g/100 mL NaOH were added, respectively. The samples were incubated for 15 min. Then, the absorbance was measured at 510 nm using a spectrophotometer. The content of total flavonoids was calculated as rutin equivalents.

Chlorogenic acid was measured according to the methods described by Jinlong Wan (2019) [85]. A total of 0.05 g of powered sample was immersed in 5 mL of methanol-0.4% phosphoric acid solution. After 24 h, they were sonicated for 30 min and centrifuged for 5 min at 16,000 g. Then the suspension was filtered through a 0.22 μm millipore membrane for HPLC equipped with a ZORBAX Eclipse XDB-C18 column (250 × 4.6 mm, 5 μm; Agilent, Santa Clara, CA, USA). Two solvent systems of the mobile phase: solvent A, acetonitrile/0.4% phosphoric acid (10:90, v:v); solvent B, 100% acetonitrile. The HPLC operating procedures was the same as that described in Jinlong Wan (2019) [85]. Chlorogenic acid concentrations of samples were calculated in relation to standard curves generated with relevant standards.

The water extracts were measured as described by Jianyong Zhang (2010) [86]. Briefly, 1.0 g of powdered sample was immersed in 150 mL hot ddH_2_O in boiling water bath for 45 min and shake once every 10 min, then vacuum filtered. Weighing chrysanthemum residues that heated in 120 °C dryer for1 h. The above process was repeated until two consecutive weighing of chrysanthemum residue mount difference was less than 0.005 g. The water extracts content (%) = (1-the weight of the last chrysanthemum residue/weight of powdered sample) × 100%.

### Measurement of N concentration

Flower samples power was digested in sulfuric acid (H_2_SO_4_) and hydrogen peroxide (H_2_O_2_), the assay of total N was performed as described previously by Liu Wei (2010) [71].

### RNA isolation and sequencing

Total RNA of chrysanthemum flowers extraction, RNA-Seq cDNA library preparation and sequencing were carried out by Shanghai Origingene Bio-pharm Technology Co. Ltd. According to the manufacturer’s protocol, total RNA from chrysanthemum flowers were extracted and purified using TRIzol reagent (Invitrogen Corp., Carlsbad, CA, USA). RNA concentration was measured by nanophotometer (Implen, Inc., Westlake Village, CA, USA). RNA integrity was assessed through the RNA Nano 6000 Assay Kit of the Bioanalyzer 2100 system (Agilent Technologies, CA, USA). Briefly, cDNA library construction was performed with the TruseqTM RNA sample prep Kit for Illumina (NEB, USA). In total six libraries of flowers under two culture system were sequenced on the Illumina Hiseq™ 2500 (Shanghai Origingene Bio-pharm Technology Co. Ltd. Shanghai, China).

### Analysis of RNA-sequence data

Using HISAT (Version: v2.1.0) [87, 88] to filter and align the reads, and mapped the clean reads with the *chrysanthemum indicum* ‘Nankingense’ gene sequence reference datasets (Chrysanthemum_genome_scaffolds_v2.0) [52]. Then, using perl scripts, the transcriptome from all samples were merged to reconstruct a comprehensive transcriptome. Based on final transcriptome, StringTie [89] and Ballgown [90] were used to estimate the expression levels of all transcripts. The differentially expressed genes were selected by R package edgeR [90], the fold differences (> = 2), statistical significance (FDR < 0.05).

### Enrichment analysis

Gene Ontology (GO) and Kyoto Encyclopedia of Genes and Genomes (KEGG) analysis were performed to obtain more detailed information about DEGs in GO terms (http://geneontology.org/) (Gene ontology consortium 2001) and metabolic pathways (https://www.genome.jp/kegg/) [91]. The DEGs in the KEGG pathways and GO analysis were enriched by ClusterProfile R packages [55]. The threshold at padj < 0.01 was determined the significant enrichment of KEGG pathways and GO terms.

### Real-time PCR analysis

To validate the gene expression, the extraction of total RNA, cDNA synthesis, and qRT-PCR were performed as previously described [92]. We chose ten DEGs involved in flavonoid synthesis genes, plant hormone signal transduction genes, photosynthesis genes, and the primer sequences were listed in Table S8. We selected chrysanthemum *GAPDH* (Gene id: CHR00001231) as a reference for gene expression analysis. We calculate the relative expression of mRNA using 2^-ΔΔCT^ equation [93, 94].

### Statistical analysis

Using SPSS software version 18.0 (SPSS Inc., Chicago, IL, USA) performed the statistical analysis of the number and dry weight of the chrysanthemum flowers, flavonoids and chlorogenic acid contents, N Concentration of the chrysanthemum flowers, as well as relative expression level and FPKM analysis.

## Supplementary Information

**Additional file 1: Figure S1.** Pearson correlation coefficients among 3 biological replications from chrysanthemum flowers under the soil system (Flower-CK-1, Flower-CK-2 and Flower-CK-3) and hydroponic system (Flower-SP-1, Flower-SP-2 and Flower-SP-3). The numbers in the scale bar stand for correlation coefficients. **Figure S2.** Principal component analysis among 3 biological replications from flowers under soil and hydroponic system. **Figure S3.** Difference of total nitrogen content in chrysanthemum flower under soil and hydroponic system. *P < 0.05, **P < 0.01.

**Additional file 2: Table S1.** GO enrichment of chrysanthemum flowers. **Table S2.** KEGG pathways enriched in the chrysanthemum flowers. **Table S3.** List of flavonoid biosynthesis-related DEGs. **Table S4.** List of Phenylpropanoid biosynthesis-related DEGs. **Table S5.** List of fatty acid and unsaturated fatty acid biosynthesis-related DEGs. **Table S6.** List of cell wall metabolism-related DEGs. **Table S7.** List of TF-related DEGs. **Table S8.** List of the quantitative primers for qRT-PCR in this study. **Table S9.** List of hormone-related DEGs. **Table S10.** List of photosynthesis-related DEGs.

## Data Availability

RNA-Seq data generated in the study have been deposited in the National center for Biotechnology Information (NCBI) under the accession codes of Bio Project ID: PRJNA744367 and SRA submission ID: SUB9968352 (https://www.ncbi.nlm.nih.gov/sra/PRJNA744367).

## Abbreviations

DEGs: Differentially expressed genes
GO: Gene Ontology
KEGG: Kyoto Encyclopedia of Genes and Genomes
FPKM: Reads mapped per 1000 bp per million sequenced reads
ABA: Abscisic acid
AUX: Auxin
JA: Jasmonic acid
BR: Brassinosteroid
GA: Gibberellin
ARF: Auxin response factor
EXP: Expansin
PE: Pectin esterase
PG: Polygalacturonase
PL: Pectate lyase
XTH: Xyloglucan endotransglycosylase/hydrolase
XYL: Xylosidase
TF: Transcription factor
qRT-PCR: Quantitative real-time PCR
