# Supplementary figures and images for: Comparison of chrysanthemum flowers grown under hydroponic and soil-based systems: yield and transcriptome analysis
Source: BMC Plant Biol. 2021 Nov 8;21:517. doi: 10.1186/s12870-021-03255-4 (PMC8574001; doi:10.1186/s12870-021-03255-4)

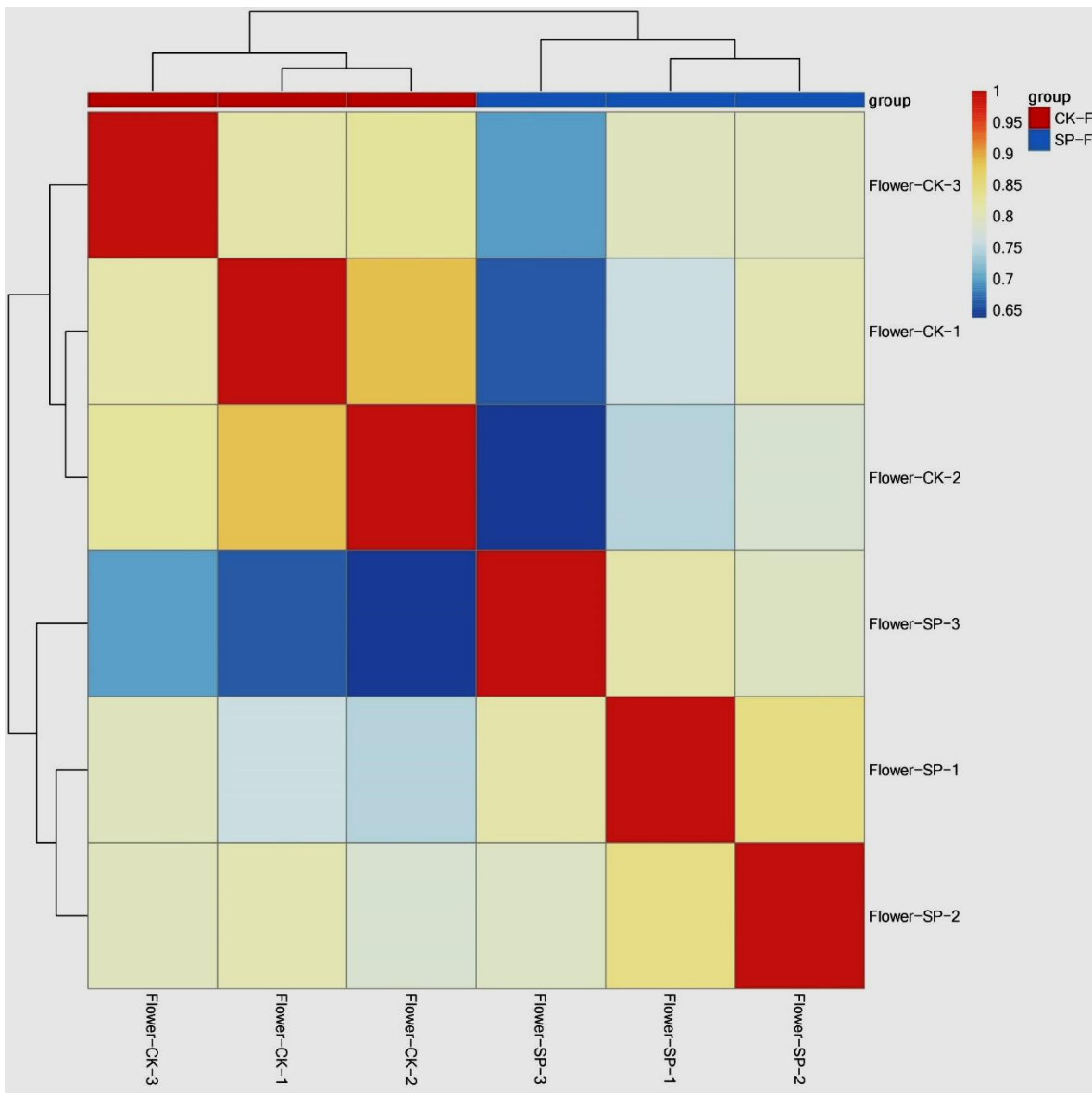

Supplement: Supplementary file 1 — Additional file 1: Figure S1. Pearson correlation coefficients among 3 biological replications from chrysanthemum flowers under the soil system (Flower-CK-1, Flower-CK-2 and Flower-CK-3) and hydroponic system (Flower-SP-1, Flower-SP-2 and Flower-SP-3). The numbers in the scale bar stand for correlation coefficients. Figure S2. Principal component analysis among 3 biological replications from flowers under soil and hydroponic system. Figure S3. Difference of total nitrogen content in chrysanthemum flower under soil and hydroponic system. *P < 0.05, **P < 0.01. [file 12870_2021_3255_MOESM1_ESM.zip › Fig S 1.pdf]

PCA 1-2-3

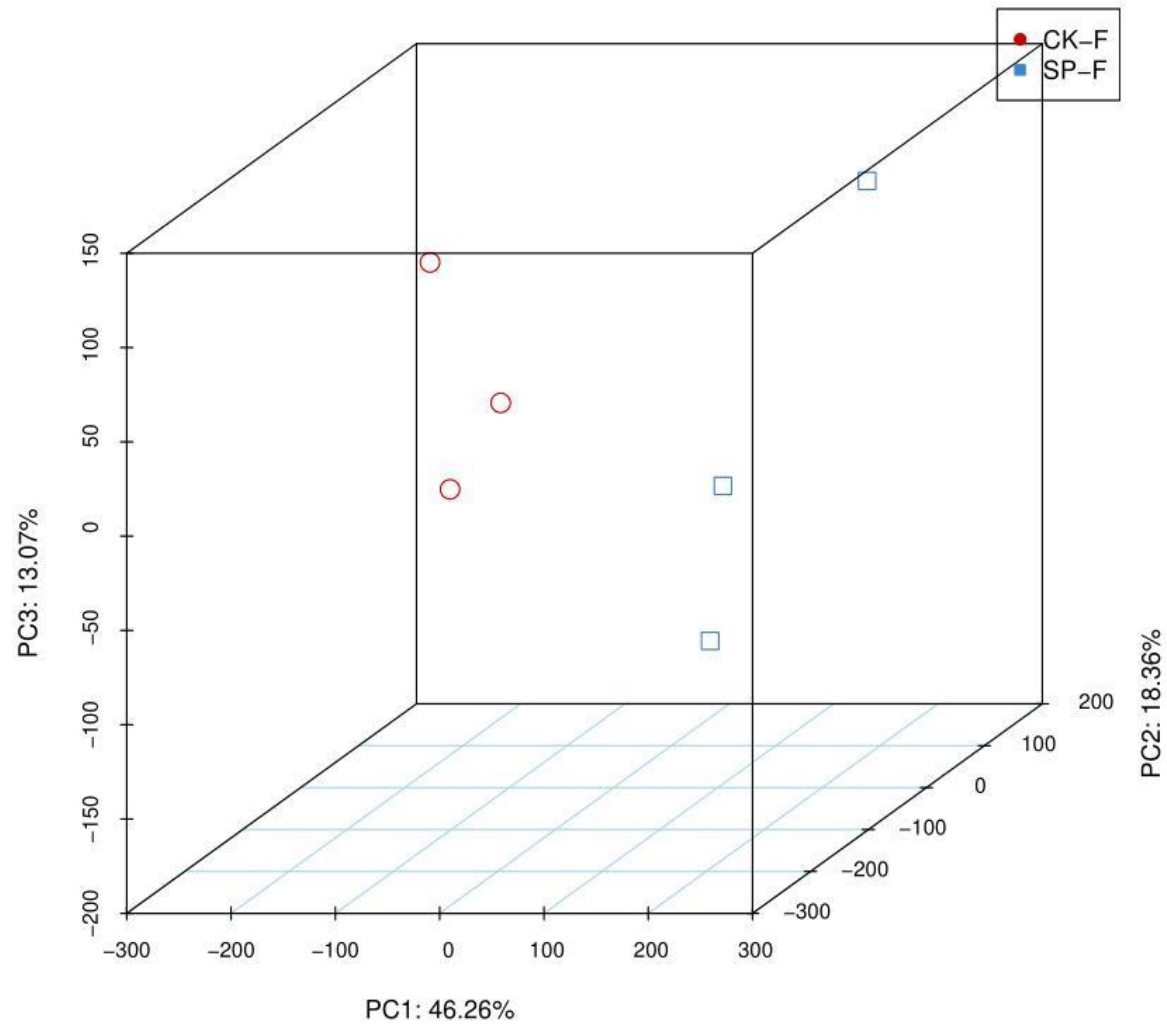

Supplement: Supplementary file 1 — Additional file 1: Figure S1. Pearson correlation coefficients among 3 biological replications from chrysanthemum flowers under the soil system (Flower-CK-1, Flower-CK-2 and Flower-CK-3) and hydroponic system (Flower-SP-1, Flower-SP-2 and Flower-SP-3). The numbers in the scale bar stand for correlation coefficients. Figure S2. Principal component analysis among 3 biological replications from flowers under soil and hydroponic system. Figure S3. Difference of total nitrogen content in chrysanthemum flower under soil and hydroponic system. *P < 0.05, **P < 0.01. [file 12870_2021_3255_MOESM1_ESM.zip › Fig S 2.pdf]

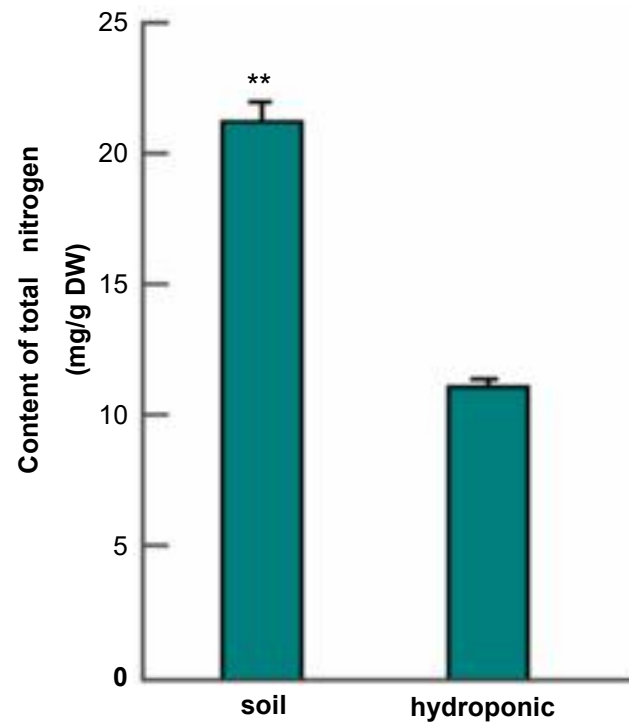

Supplement: Supplementary file 1 — Additional file 1: Figure S1. Pearson correlation coefficients among 3 biological replications from chrysanthemum flowers under the soil system (Flower-CK-1, Flower-CK-2 and Flower-CK-3) and hydroponic system (Flower-SP-1, Flower-SP-2 and Flower-SP-3). The numbers in the scale bar stand for correlation coefficients. Figure S2. Principal component analysis among 3 biological replications from flowers under soil and hydroponic system. Figure S3. Difference of total nitrogen content in chrysanthemum flower under soil and hydroponic system. *P < 0.05, **P < 0.01. [file 12870_2021_3255_MOESM1_ESM.zip › Fig S3.pdf]
